# New psychoactive substances in roadway crash victims in California

**DOI:** 10.3389/ftox.2025.1572324

**Published:** 2025-06-25

**Authors:** Roy Gerona, Daniel Tomer, Donovan Nielsen, Allyson C. Sage, Deborah French, Juliana Tolles, James Alan Chenoweth

**Affiliations:** ^1^ Department of OB/Gyn and Reproductive Sciences, University of California, San Francisco, San Francisco, CA, United States; ^2^ Department of Emergency Medicine, University of California, Davis, Davis, CA, United States; ^3^ Department of Laboratory Medicine, University of California, San Francisco, San Francisco, CA, United States; ^4^ Department of Emergency Medicine, Harbor-UCLA, Los Angeles, CA, United States

**Keywords:** impaired driving, roadway crashes, new psychoactive substances, fentanyl analogs, designer benzodiazepines, mitragynine, LC-QTOF/MS, California

## Abstract

**Background:**

Motor vehicle crashes (MVCs) are a major global health concern. While alcohol continues to be a significant contributor to MVCs, the role of illicit and prescription drugs has increased in the last 4 decades. Moreover, the proliferation of new psychoactive substances (NPS) in the United States since 2010 has reshaped recreational drug use. Despite this, its contribution to MVCs has not been systematically evaluated. In this study, we report the prevalence of NPS in roadway crash victims in California.

**Methods:**

Serum samples from 1000 roadway crash victims were collected and analyzed using liquid chromatography-quadrupole time-of-flight mass spectrometry (LC-QTOF/MS) against a comprehensive database of 1314 drugs, including 1008 NPS, and quantitative analysis was performed using isotope dilution. Alcohol was quantified in an autoanalyzer using an enzymatic method employing alcohol dehydrogenase.

**Results:**

Eight NPS (detection frequency = 26) were confirmed and quantified in 17 cases. Like current nationwide NPS surveillance studies, bromazolam, para-fluorofentanyl, and mitragynine were most frequently detected. NPS were detected in polypharmacy use, with traditional recreational drugs such as fentanyl, methamphetamine, and delta-9 THC most frequently co-detected. The serum geometric means detected for bromazolam (5.41 ng/mL; range: 0.22–26.59), para-fluorofentanyl (0.45 ng/mL; range: 0.28–2.02) and mitragynine (7.02; range: 0.55–90.55) were lower than those reported for overdose and death cases.

**Discussion:**

This study is the first to report quantitative levels of multiple NPS and multiple NPS classes in a large US roadway crash survey, with the high detection of CNS depressants and their co-occurrence with traditional recreational drugs highlighting the need for expanded NPS testing, roadside testing strategies, and guidelines for determining drug-induced impairment; the quantitative data may be valuable in establishing these guidelines.

## Introduction

Motor vehicle crashes (MVCs) are a leading cause of morbidity and mortality. The United Nations estimates that one person dies in a road crash every 24 s. That is roughly 1.35 million people each year (United Nations, 2024). While alcohol has long been established to be a significant contributor to MVCs, there has been interest in the role of illicit and prescription drugs in the last 4 decades. While there are several ways to classify drugs, that is, chemical makeup, origins, and legal status among others, for the purposes of this paper, drugs will be classified within general categories based on what is most relevant to the pharmacologic mechanisms that facilitate impaired driving. Psychoactive drugs including hallucinogens (cannabis, LSD), central nervous system (CNS) stimulants (methamphetamine, cocaine), CNS depressants (opioids, benzodiazepines), dissociative anesthetics (phencyclidine, ketamine), and sedating prescription drugs (some antihistamines, antipsychotics and antidepressants) can contribute to impaired driving which may lead to MVCs.

Drugs can impair driving through a combination of different mechanisms. These include impairing cognitive processes that are critical in driving, such as focus, attention, and decision-making. Psychoactive drugs can also compromise psychomotor skills and coordination, which are essential in observing correct lane position and speed while driving. Some drugs can lower inhibition, which can lead to aggressive driving, speeding, and a loosened attitude toward following traffic rules. Other drugs and medications can cause sedation and fatigue that may compromise alertness and delay reaction time to urgent and critical driving situations (Riedel et al., 1998).

Surveys and studies on the involvement of drugs in impaired driving and MVCs started in the 1980s (Williams et al., 1985). Two surveys conducted on car drivers killed in traffic crashes in Los Angeles in 1985–86 and 1987–88 detected drugs in 30% (total sample size (N) = 102) and 12% (N = 492) of the drivers, respectively (Budd et al., 1989). Cannabis and cocaine were the most detected drugs and in at least 50% of the cases that tested positive for drugs, alcohol was co-detected. Nationwide, a review of data from the Fatality Analysis Reporting System, a survey of MVCs resulting in at least one fatality on US public roads, showed a steady increase in drug prevalence in MVCs from 18.1% in 1999 (N = 6,686) to 29.1% in 2010 (N = 7,032). Cocaine and cannabis were the most reported drugs (Rudisill et al., 2014; Brady and Li, 2014). However, prescription drugs as a broad category were more prevalent than cannabis, cocaine, methamphetamine and various Schedule I drugs. Among the prescription drugs, the detection of oxycodone, hydrocodone, and alprazolam registered significant and steady increases starting in 1999 (Rudisill et al., 2014). More than 50% of drivers who tested positive for cannabis (N = 998) or cocaine (N = 145) were also using alcohol at the time of the MVC (Wilson et al., 2014). Polypharmacy in the cases also increased from 4.5% in 1999 (N = 6,686) to 7.3% in 2010 (N = 7,032) (Brady and Li, 2014). Additionally, a recent study by the National Highway Traffic Safety Administration (NHTSA) reported the presence of potentially impairing drugs (both illicit and pharmaceutical) in 54.4% of injured drivers (N = 6,382) and 68.8% of driver fatalities (N = 897, Thomas et al., 2022).

The rapid rise of new psychoactive substances (NPS, aka designer drugs) in the recreational drug market that started in 2010 complicated drug testing in clinical and forensic laboratories. The rapid molecular evolution of the composition of the drug products containing NPS required a paradigm shift in drug testing (Gerona and French, 2022). Most are missed in urine drug screens and the traditional targeted method used in drug confirmation is unable to cope with the rapid introduction of new NPS. Thus, NPS are often missed unless non-targeted analysis facilitated by high-resolution mass spectrometry (HRMS) is incorporated into a laboratory’s drug testing workflow. Unfortunately, very few laboratories are able to implement HRMS testing due to the high cost of the platform and the specialized expertise it requires that is not commonly available (Gerona and French, 2022; Wu et al., 2012). Thus, NPS are underreported in the context of impaired driving and MVCs.

The recognition of NPS as an important component of drug testing in impaired driving and MVCs was reflected in the inclusion of fentanyl analogs as Tier 2 drugs in the updated 2017 recommendations for toxicological investigation of driving under the influence of drugs (DUID) and MVC cases (Logan et al., 2018). Inclusion of other NPS classes such as synthetic cannabinoids, synthetic cathinones and designer benzodiazepines among others followed in the 2021 recommendations (D’Orazio et al., 2021). Tier 2 drugs are those that are less frequently encountered or require more advanced instrumentation like HRMS (Logan et al., 2013). Despite this, very few surveys on DUID and MVC cases in the United States have reported the detection of NPS. A handful of reports have been published in Europe. NPS, primarily synthetic cathinones, were detected in 33 out of 391 oral fluid samples (8.4%) collected from suspected DUID cases in France and Belgium in 2016 (Richeval et al., 2019). In another study, stimulant NPS (3%–8%) and synthetic cannabinoids (20%–22%) were detected in DUID case samples positive for drugs collected between 2016 and 2018 in Hungary (Institóris et al., 2022).

In this study, we report the NPS detected in samples collected from roadway crash victims in northern and southern California in 2024. A roadway crash is defined as a crash involving a motor vehicle, bicycle, mini-mobility device, or pedestrian struck by a motor vehicle on a public roadway. A motor vehicle crash is a subtype of roadway crash that is limited to mechanically or electrically powered devices not operated on rails, i.e., motor vehicles. To the best of our knowledge, this is the first formal report of multiple NPS and NPS classes quantified in a large survey of roadway crash cases in the United States.

## Materials and methods

### Eligibility and enrollment

All patients 18 and older presenting to two urban trauma centers in California within 6 h of a roadway crash who were undergoing a blood draw as part of their routine Emergency Department (ED) care were eligible for inclusion. Patients were excluded if they were not undergoing a blood draw as part of their ED care, if a blood draw was performed without collecting blood for drug and alcohol testing, if the initial blood draw occurred more than 6 h from the time of the crash, or if the patient was in police custody at the time of presentation or was taken into custody during the first 24 h of hospitalization. Enrollment began on 2 January 2024 and is ongoing. This study was approved by the local institutional review boards (IRB number 2056749) with a full waiver of consent and received a certificate of confidentiality from the National Institutes of Health.

### Sample collection, preparation, and storage

Five mL of blood was collected in both a red top tube and a lithium heparin tube by the bedside nurse during a routine lab draw. Red top tubes were then centrifuged at 1300 relative centrifugal force (RCF) for 10 min and serum was aliquoted into cryotubes which were then refrigerated at 1°C–6°C until shipped to the UCSF Clinical Toxicology and Environmental Biomonitoring Laboratory. Lithium heparin tubes were centrifuged and frozen at −20°C until shipped. Shipments occurred once per week. Alcohol analysis was performed at UCSF Health Clinical Laboratories.

### Sample analysis

#### Drugs

We analyzed drugs in serum samples using a modification of our published method (Lung et al., 2016). Briefly, a mixture of 14 internal standards was added to each serum sample for a final concentration of 25 ng/mL. The sample (0.25 mL) was prepared for analysis by protein precipitation using 0.75 mL 3:1 acetonitrile: methanol. The extract obtained was reconstituted in 0.25 mL 10% acetonitrile prior to injection into an Agilent liquid chromatograph (LC) 1260 attached to an Agilent quadrupole time-of-flight mass spectrometer (QTOF/MS) 6550. Analytes were separated by gradient elution chromatography using a reverse phase Agilent Poroshell 120 EC-C18 column (2.1 × 100 mm, 2.7 µm). Water with 0.05% formic acid and 0.1% 5 M ammonium formate was used as mobile phase A (MPA) and acetonitrile with 0.05% formic acid as mobile phase B (MPB). The gradient used for analyte separation started with 5% MPB from 0 to 1.5 min, 30% MPB from 1.5 to 4.5 min, 70% MPB from 4.5 to 7.5 min, and 100% MPB from 7.5 to 10 min at a flow rate of 0.5 mL/min. An injection volume of 2.5 µL was used. The column compartment temperature was set to 50°C, and the autosampler temperature was set to 4°C.

Mass spectrometry was performed using the TOF/MS mode in one run followed by a QTOF/MS run using auto MS/MS data acquisition. An electrospray ionization source in the positive and negative modes was used to ionize analytes in the extract. The QTOF/MS was run under the following conditions: gas temperature at 225°C; sheath gas temperature at 350°C; drying gas flow at 14L/min; sheath gas flow at 11L/min; nebulizer pressure at 14 psi; voltage cap at 3000 V (positive mode) or −2,500 V (negative mode); and, nozzle voltage at 500 V (positive mode) or −1,500 V (negative mode). Data acquisition was run at 2 GHz in extended dynamic range mode. A TOF-MS scan across the range of 75–1000 m/z was collected at high resolution. Using the Auto MS/MS mode (information-dependent acquisition), a product ion scan (MS/MS) of the three most abundant peaks at high resolution was triggered each time a precursor ion with an intensity of ≥500 counts per second was generated in the TOF-MS scan; active exclusion of previously selected peak was held for 0.5 min. The MS/MS scan range used was 50–1000 m/z.

Quantitative analysis of confirmed NPS was accomplished using isotope dilution. Each sample was run along with a 12-point calibration curve using the LC-TOF/MS analysis method described above. A mixture of 14 internal standards (11-nor-9-carboxy-delta-9-THC-d3, amiodarone-d4, atropine-d3, cocaine-d3, delta-9-THC-d9, doxepin-d3, fentanyl-d5, hydromorphone-d6, JWH-015-d7, loratadine-d4, MDMA-d5, morphine-d6, oxycodone-d6, paroxetine-d6) with retention times that bracket the retention times of all the drugs in the laboratory’s comprehensive drug database was used.

#### Alcohol

Alcohol was quantified in plasma samples using an enzymatic method employing alcohol dehydrogenase that converts any alcohol in the sample to acetaldehyde (Abbott Diagnostics). The enzymatic reaction is monitored spectrophotometrically at 340 nm. If the ethanol concentration in the sample is < 0.01 g/dL it is reported as negative. If the concentration is ≥0.01 g/dL, the quantitative concentration is reported.

#### Data analysis

Drug screening was performed on the total ion chromatogram (TIC) obtained from LC-TOF/MS analysis using the Agilent MassHunter Qualitative Analysis software and a comprehensive database consisting of 1314 drugs, of which 1008 are NPS. This consists of alkylamines (3), aminoindanes (6), amphetamines (29), anabolic steroid (1), arylcyclohexylamines (17), designer benzodiazepines (38), lysergamides (2), new synthetic opioids (296), phenethylamines (28), plant-derived opioids (2), piperazines (9), piperidines (9), synthetic cannabinoids (481), synthetic cathinones (67), and tryptamines (20). The database also includes traditional recreational drugs (54), prescription and over-the-counter drugs (215), precursors (5), additives (5) and impurities (1), dietary supplement stimulants (23), other dietary supplement ingredients (1), and nicotine and its metabolites (2).

To screen for presumptive matches, the following criteria were used: mass error within 10 parts per million (ppm), retention time match within 0.15 min, and a target score ≥70 (an indicator of mass spectral isotopic abundance and spacing or isotopic pattern matches). For confirmation of presumptive positive matches, data from the LC-QTOF/MS run was analyzed. A spectral library match score ≥70 (indicator of fragment ion data match) was imposed for confirmation.

The quantitative levels of confirmed NPS were measured using the Agilent MassHunter Quantitative Analysis software. The isotopologue with the closest retention time to the analyte of interest was used as internal standard.

## Results

While sample collection is still ongoing, the first 1000 cases included in our present analysis were collected between 2 January 2024 and 25 July 2024. These roadway crash victims originated from Los Angeles and Sacramento, California. NPS was confirmed in 17 cases (1.7%). Ten of the cases were from Sacramento while the remaining seven were from Los Angeles.

The average age of roadway crash victims with confirmed NPS was 34 (range:18–66; median: 38). Of the 17 cases, 11 were males. Seven were passengers, five were drivers, three were bicyclists with two being on electric bikes, one pedestrian struck by a motor vehicle, and one in mini-mobility device. The types of crashes involved in the cases include single vehicle (8), multi-vehicle (5), auto vs. bicycle (2), auto vs. pedestrian (1), and bicyclist (1) (Table 1).

**TABLE 1:** Characteristics of seventeen NPS-positive roadway crash cases including specific drugs detected. Source locations were not included to protect patient confidentiality*.

Age/Gender/Type of crash	NPS	Conc’n (ng/mL)	Traditional recreational drug (ng/ML)	Precursor, additive, impurity	Over-the-counter or prescription drug	Ethanol (g/dL)
33 y/o Male Driver Single Vehicle Crash	para-Fluorofentanyl	0.28	Negative	4-ANPP Lidocaine	Acetaminophen	Negative
49 y/o Male Passenger Multi-vehicle crash	7-Hydroxymitragynine Mitragynine	8.92 90.55	Hydrocodone (62)	Negative	Duloxetine	Negative
27 y/o Female Driver Mini-Mobility Device	Bromazolam N-methyl norfentanyl	26.59 0.83	Beta-hydroxy fentanyl (2.4) Fentanyl (28.1) Norfentanyl (35.4)	4-ANPP	Acetaminophen	Negative
33 y/o Female Bicyclist Auto vs. Bicycle	Acetyl fentanyl para-Fluorofentanyl Protonitazene	0.55 0.28 0.55	Beta-hydroxy fentanyl (1.6) Norfentanyl (9.9) Methamphetamine (94.4)	4-ANPP Lidocaine N,N-Dimethylamphetamine	Acetaminophen	Negative
21 y/o Male Passenger Single Vehicle Crash	Etizolam	1.21	Delta-9-THC (2.7)	Negative	Alprazolam Diphenhydramine Promethazine	Negative
18 y/o Female Passenger Single Vehicle Crash	Bromazolam	10.62	Benzoylecgonine (60.8) Cocaine (2.7)	Negative	Negative	Negative
32 y/o Male Passenger Single Vehicle Crash	Bromazolam para-Fluorofentanyl N-methylnorfentanyl	0.22 0.28 0.55	Benzoylecgonine (55) Beta-hydroxy fentanyl (5.5) Norfentanyl (12) Methamphetamine (127)	4-ANPP	7-aminoclonazepam Clonazepam Alprazolam	Negative
42 y/o Male Driver Single Vehicle Crash	para-Fluorofentanyl	2.02	Benzoylecgonine (3.3) Cocaine (0.5) Fentanyl (3.7) Norfentanyl (0.3) Methamphetamine (91.5)	4-ANPP	Negative	Negative
23 y/o Male Driver Single Vehicle Crash	Mitragynine	0.55	Negative	Negative	Negative	Negative
23 y/o Male Driver Multi-Vehicle Crash	Alpha-hydroxy bromazolam Bromazolam	10.58 1.08	4-Hydroxymethamphetamine (0.1) Methamphetamine (300) Beta-hydroxy fentanyl (2.9) Norfentanyl (2.8) Delta-9-THC (8.6)	4-ANPP Quinine	Acetaminophen	Negative
22 y/o Female Passenger Single Vehicle Crash	Bromazolam	17.01	Beta-hydroxy fentanyl (0.5) Fentanyl (3.2) Norfentanyl (2.8) Methamphetamine (0.6)	Quinine	Negative	Negative
54 y/o Male Bicyclist E-Bike Crash	Xylazine	4.10	11-nor-9-carboxy-delta-9-THC (70) Delta-9-THC (25.6)	Negative	1-(3-Chlorophenyl) piperazine Trazodone Acetaminophen Carbamazepine Desmethyldoxepin Doxepin Haloperidol Oxcarbazepine Quetiapine	0.35
34 y/o Male Driver Single Vehicle Crash	7-Hydroxymitragynine Mitragynine	33.09 88.41	Fentanyl (68.9) Norfentanyl (1.6)	4-ANPP	Negative	Negative
66 y/o Female Passenger Multi-Vehicle Crash	Mitragynine	0.55	Hydrocodone (3.6)	Negative	1-(3-Chlorophenyl) piperazine Trazodone	Negative
33 y/o Male Pedestrian Struck by Vehicle	Bromazolam	13.53	Beta-hydroxy fentanyl (0.3) Fentanyl (3.6) Norfentanyl (3.4) Methamphetamine (18.6)	4-ANPP	Negative	Negative
29 y/o Female Bicyclist E-Bike vs. Vehicle	para-Fluorofentanyl Acetyl fentanyl	0.28 0.55	Beta-hydroxy fentanyl (7.8) Fentanyl (47.1) Norfentanyl (27.5) 4-Hydroxymethamphetamine (1.1) Methamphetamine (504) Amphetamine (76.2)	4-ANPP Lidocaine	Acetaminophen	Negative
35 y/o Male Passenger Multi-Vehicle Crash	Bromazolam	8.87	11-nor-9-carboxy-delta-9-THC (61.5) Delta-9-THC (1.5) Methamphetamine (15.6)	Negative	Negative	Negative

*The limits of quantification (LOQ) for the analytes (in ng/mL) are as follows: 4-Hydroxymethamphetamine (0.1); 7-Hydroxymitragynine (0.39); 11-nor-9-carboxy-delta-9-THC (60); Acetyl fentanyl (0.39); Alpha-hydroxy bromazolam (0.2); Amphetamine (60); Benzoylecgonine (0.39); Beta-hydroxy fentanyl (0.2); Bromazolam (0.2); Cocaine (0.1); Delta-9-THC (1); Etizolam (0.78); Fentanyl (0.78); Hydrocodone (1.6); Methamphetamine (0.39); Mitragynine (0.39); N-Methyl norfentanyl (0.2); Norfentanyl (0.2); para-Fluorofentanyl (0.2); Protonitazene (0.39); Xylazine (1.56). Details of the analytical method used to quantify the analytes are beyond the scope of this paper but they can be requested from the corresponding author.

Of the 1000 cases, 290 were positive for at least one traditional recreational drug (TRD) or NPS (29%). Of the 288 that were positive for TRDs, 15 have NPS (4.9%). There were eight unique NPS detected with an overall detection frequency of 26. Only CNS depressants were detected, including designer benzodiazepines, new synthetic opioids, and the plant-derived opioid, mitragynine. Bromazolam (detection frequency, DF = 7) was the most detected, followed by para-fluorofentanyl (DF = 4) and mitragynine (DF = 3) (Table 1). The five other NPS detected includes acetyl fentanyl, N-methyl norfentanyl, protonitazene, etizolam and xylazine. The quantitative levels of these NPS measured in serum samples are given in Table 1. In one case three NPS were detected, while in six, two NPS were detected.

All but one case also had one or more traditional recreational drugs (TRD), of which fentanyl (DF = 9), methamphetamine (DF = 9), and delta-9-THC (DF = 4) were the most detected. Cocaine was detected in 2 cases while the inactive cocaine metabolite, benzoylecgonine, was detected in 3 cases (Table 1). In nine cases the precursor for fentanyl, ANPP, was detected. Two adulterants, quinine and lidocaine, were detected in two and three cases, respectively. The impurity obtained during methamphetamine synthesis, N,N-dimethylamphetamine, was detected in one case.

Sixteen of the seventeen cases tested negative for ethanol. The sole sample that tested positive for alcohol had a concentration of 0.35 g/dL (Table 1).

## Discussion

The analysis of the first 1000 cases indicates that NPS in roadway crashes is a cause for concern in the United States. Eight NPS with a detection frequency of 26 were confirmed in 17 cases. Importantly, the NPS detected at the highest frequencies (bromazolam, para-fluorofentanyl, and mitragynine) are all categorized as CNS depressants. These drugs reduce the activity of the brain, resulting in sedation, drowsiness, and impaired cognitive and motor functions (Hetland and Carr, 2014). Previous studies have found an association between heightened crash risk and the type of drug used while driving with depressants resulting in the highest risk (Li et al., 2013). Benzodiazepine users have been found to be up to 80 percent more likely to be involved in a motor vehicle crash compared to non-users (Dassanayake et al., 2011). Bromazolam, the most frequently detected NPS in this study, is a designer benzodiazepine. This NPS is the brominated counterpart to the chlorinated drug alprazolam, most popularly prescribed as Xanax (Zawilska and Wojcieszak, 2019). *In vitro* studies predicted bromazolam to be a non-subtype selective agonist at the benzodiazepine site of GABA-A receptor with binding affinities of 2.81 nM at the α1 subtype, 0.69 nM at α2, and 0.62 nM at α5 (Clayton et al., 2015). Bromazolam was initially reported in a retrospective analysis of DUID cases submitted by law enforcement agents that tested positive for designer benzodiazepines between 2017 and 2021. Bromazolam was one of eight benzodiazepines included in 1145 designer benzodiazepine detections in 805 blood samples. However, it only accounted for five detections (0.4%) (Papsun et al., 2023). The relatively high detection of bromazolam in our cohort is consistent with recent research reporting 98 out of 52,585 (0.19%) impaired driving cases from 2021 to 2023 in which the driver tested positive for bromazolam via LC-TOF/MS and/or immunoassay screenings (Bierly et al., 2024). Our higher detection frequency of 0.7% is consistent with the rise of bromazolam as the most detected NPS in the US Drug Enforcement Administration Toxicology Testing Program (DEA TOX) that started in 2023. In almost all of these cases, bromazolam was detected along with fentanyl suggesting either their intentional co-ingestion or the co-presence of fentanyl and bromazolan in drug products, consistent with the increasing prevalence of “benzo dope”.

The second most detected NPS in our study, para-fluorofentanyl (pFF), was the most frequently detected NPS in the United States from 2021 until it was overtaken by bromazolam in 2023 (Trecki et al., 2022; U.S. Drug Enforcement Administration, Diversion Control Division, 2023b). This NPS was sold briefly on the US black market in the early 1980s, which served as the impetus for the introduction of the Federal Analog Act (Henderson, 1988). It made its comeback in 2020 when it was frequently detected as a fentanyl adulterant. pFF has been detected previously in a similar study conducted by the National Highway Traffic Safety Administration (NHTSA) between 2021 and 2022; pFF was detected 13 times over 2 years in 7,279 roadway users (0.18%) across seven different states (Thomas et al., 2022). One explanation for our higher detection frequency (0.4%) of pFF may be that some laboratories that performed the analyses in the NHTSA study do not have the ability to detect pFF.

Mitragynine, the primary psychoactive ingredient of Kratom, was the third most detected NPS in our study. Although Kratom was first brought to the United States in the 1980s, its recreational use only gained traction in 2010 (Post et al., 2019). Kratom has a stimulant effect in low doses, opioid-like effects in moderate to high doses, and sedative effects in very high doses (Prozialeck et al., 2012). Mitragynine was first reported in a DUI case in the United States in 2018 (Wright, 2018). The detection frequency in our cases (0.3%) is consistent with a previous retrospective study on the detection of mitragynine in DUID cases in Orange County, California between 2017 and 2019. In the 25,398 cases evaluated, mitragynine was detected in 60 cases (0.24%) (Kedzierski and Mata, 2023). The other NPS detected in our cases- N-methyl norfentanyl, acetyl fentanyl, protonitazene, etizolam, xylazine-have also been reported in the United States in the last 5 years (United States Drug Enforcement Administration, 2020; U.S. Drug Enforcement Administration, Diversion Control Division, 2023a).

**FIGURE 1:**
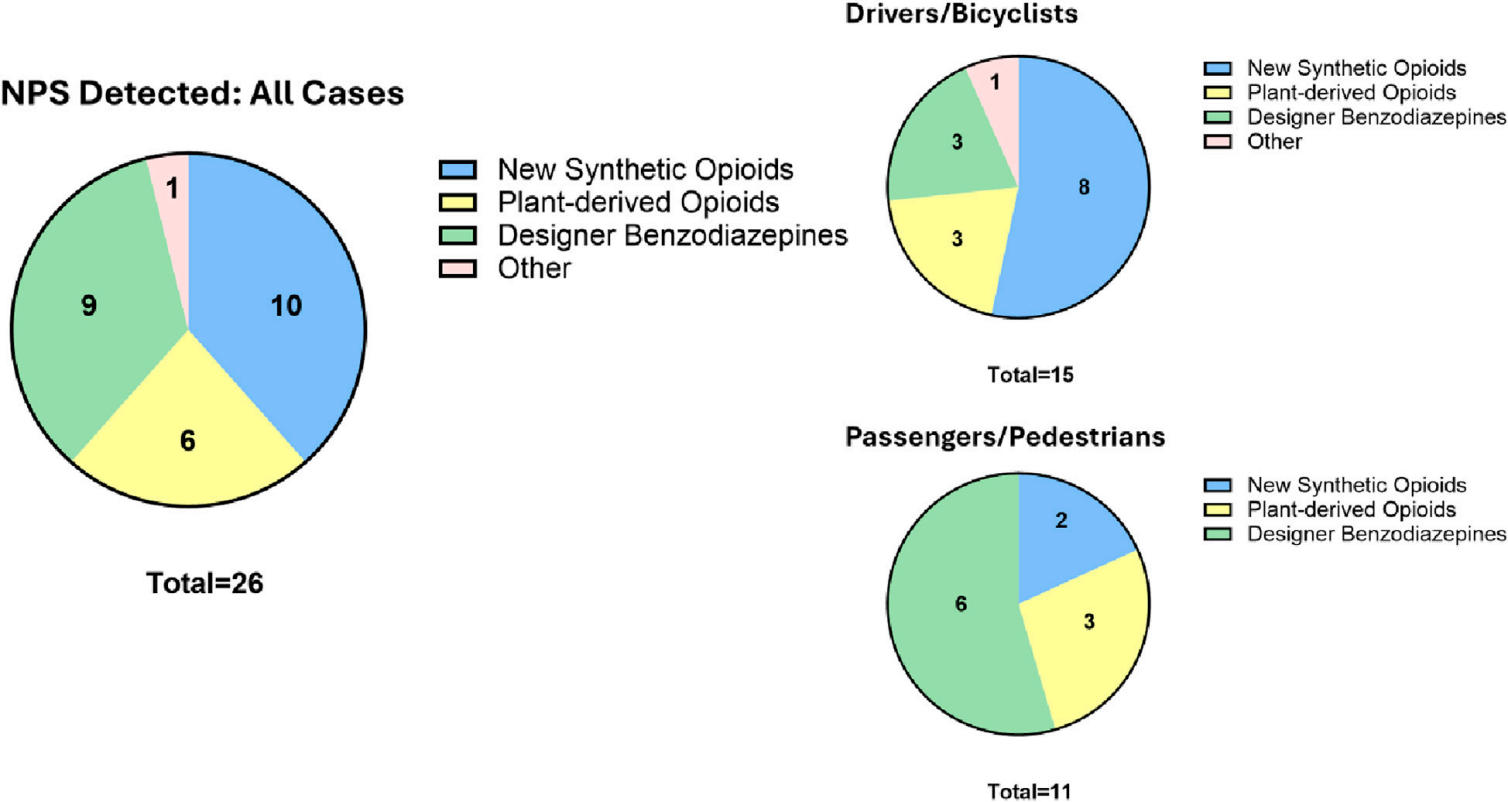
Detection frequency of NPS parent compound and metabolite by drug class and position in crash.

Five other NPS (3 new synthetic opioids, 1 designer benzodiazepine, xylazine) were quantified in six cases. As a class, opioids were detected the most (16, 62%) in the entire cohort (Figure 1); notably, new synthetic opioids were detected almost twice as much as plant-derived opioids. When the position in the crash was considered, opioids overwhelmingly predominated (11 of 15, 73%) among drivers and bicyclists. Interestingly, designer benzodiazepines (6, 55%) and opioids (5, 45%) were almost equally detected in passengers and pedestrians. Of note, designer benzodiazepines were detected twice as much in passengers compared to drivers. However, with the limited number of NPS-positive cases detected so far in our study, it is hard to ascribe any meaning yet to these observations. The study is ongoing and with more NPS cases accumulating in our second year, a more representative sampling would soon be available.

While alcohol-impaired driving continues to have the most detrimental impact on traffic safety (Thomas et al., 2022), polysubstance abuse-impaired driving is a significant contributor. Under the DRUID project, Hels and colleagues found that the odds of being seriously injured in a motor vehicle collision are 28 times higher for drivers who test positive for alcohol with other drugs and 8 times higher for drivers who tested positive for more than one nonalcohol drug compared to those who tested negative. These numbers are even higher (31x and18x, respectively) for fatally injured drivers (Hels et al., 2011).

The percentage of drivers using multiple drugs has increased from 32.6 percent in 1993 to 45.85 percent in 2010 (Wilson et al., 2014). There are several factors to account for this, including but not limited to an increase in prescription drug use (Kantor et al., 2015) and an increase in NPS supply and use in the United States. This is consistent with our data, as all but one case of NPS detection involved polypharmacy. Fifteen of the 17 cases have at least one traditional recreational drug with fentanyl detected in 9 cases. NPS are commonly co-detected with TRDs due to co-existence in drug preparations and/or because users combine different pharmacological classes of drugs they abuse.

The current fentanyl epidemic in the United States started in 2015, and since then, fentanyl has been detected as an adulterant in common drugs of abuse, initially in heroin and later in stimulants like methamphetamine and cocaine. Seven of the fentanyl detections have either methamphetamine or cocaine. Additionally, NPS are also added to fentanyl drug preparations. The majority of the bromazolam and pFF detections in NPS surveillance in the United States are co-detected with fentanyl (U.S. Drug Enforcement Administration, Diversion Control Division, 2023a; U.S. Drug Enforcement Administration, Diversion Control Division, 2023b; U.S. Drug Enforcement Administration, Diversion Control Division, 2023c; U.S. Drug Enforcement Administration, Diversion Control Division, 2023d; U.S. Drug Enforcement Administration, Diversion Control Division, 2023e). This is reflected in our cases; fentanyl was detected in five of seven and four of five cases of bromazolam and pFF cases, respectively. The combination of these depressants undoubtedly exacerbates the impact of these drugs on driving. In the two cases in which bromazolam was found without fentanyl, methamphetamine was detected. Although the combination of drugs with opposing pharmacological effects may be antagonistic in some cases, both stimulants and depressants, on their own, impact driving. More importantly, it is difficult to determine whether the effects each class has on driving are mitigated by their combination. And, even if this is the case, obtaining the specific proportion required for one drug to antagonize another may be challenging to precisely achieve and may vary from one person to another.

Furthermore, our research provides a unique insight into not just the identification of NPS detected in roadway crashes, but their quantitative levels too. Previous studies primarily report qualitative data on NPS; in a few studies, quantitative data for a specific NPS like bromazolam was presented (Bierly et al., 2024; Papsun et al., 2023). Table 1 shows the quantitative levels of all eight NPS found in roadway crash victims in our study. While there is lack of consensus regarding the correlation between drug concentration and degree of impairment due to factors such as tolerance or drug-drug interactions (Reisfield et al., 2012), there are proposed cut-offs for psychoactive substances that have clear concentration dependent cognitive impairment (Blandino et al., 2022). Quantitative drug levels may be able to provide a window into the potential degree of intoxication for those involved in the crashes. This can, in turn, provide a clue to the degree of impairment that can be ascribed to drug levels detected. A cutoff blood alcohol level has been established to objectively determine whether a motorist may be experiencing alcohol-induced driving impairment. The same has not yet been established for any other drugs including common drugs of abuse, e.g., cannabis. Hence, the NPS levels reported in our study can provide initial data that may be useful in establishing guidelines for determining NPS-induced drug impairment. The geometric mean of serum levels that we measured for bromazolam was 5.41 ng/mL (range: 0.22–26.59 ng/mL). These levels are lower than those reported in overdose cases (12.34 ng/mL) and death cases (25.39 ng/mL) by the DEA TOX program in 2023. This is expected as overdose patients are incapacitated, making driving highly unlikely. However, the wide range of values observed in roadway crash victims likely reflects differences in drug tolerance. For pFF, the geometric mean observed in roadway crash victims was 0.45 ng/mL (range: 0.28–2.02), whereas in DEA TOX it was 5.26 ng/mL (U.S. Drug Enforcement Administration, Diversion Control Division, 2023b; U.S. Drug Enforcement Administration, Diversion Control Division, 2023c; U.S. Drug Enforcement Administration, Diversion Control Division, 2023d; U.S. Drug Enforcement Administration, Diversion Control Division, 2023e). No comparison to overdose cases can be made as almost all pFF cases in DEA TOX were deaths. This and the lower concentrations observed for pFF associated with roadway crashes and deaths may suggest its stronger potency in these cases. Alternatively, because almost all pFF cases also involve fentanyl, this may be suggestive of the synergistic effects of the two drugs.

Our work is the first to conduct a comprehensive targeted analysis of more than 1000 NPS, in combination with suspect and non-targeted screening in roadway collisions across a large population. While there have been reviews across several countries in Europe such as Spain, Germany, France, and Belgium (Fels et al., 2020; Richeval et al., 2019; Soria, 2018; Wille et al., 2018), in the United States there only exist case reports for NPS in individual drivers (Fassette and Martinez, 2016; Lemos, 2014; Rajotte et al., 2017), larger studies (n = 9,569) with limited NPS testing (only synthetic cannabinoids and cathinones) and detection frequency (n = 2, <0.1% detection frequency) (NTSB, 2022), and retrospective analyses for specific NPS like bromazolam and mitragynine in large cohorts (Bierly et al., 2024; Kedzierski and Mata, 2023; Papsun et al., 2023). Counterintuitively, the United States also experiences some of the highest rates of NPS use. The reasons for why this may be the case center around regulation and policy frameworks. The United States lacks an organized federal body akin to the European Monitoring Center for Drugs and Drug Addiction (EMCDDA), which is focused on tracking the prevalence of NPS in public health that also looks specifically at road safety. This allows for a more coordinated and unified effort in which laws and testing protocols around NPS are harmonized, whereas in the United States federal and state jurisdiction disputes can complicate the implementation of consistent policies. Furthermore, there are specific policies, such as the EU Drug Driving Directive (2015/413) which targets drug-impaired driving, including NPS. There is also a larger extent and wider range of testing infrastructure in Europe including monitoring tools such as syringe residue and wastewater analysis, drug product and biological sample testing through the European Drug Emergencies Network, drug checking services, and expanded surveys (Wood and Dargan, 2020). In the United States, attempts at improving regulation through the Controlled Substances Analogue Enforcement Act (1986) as well as the Synthetic Drug Abuse Prevention Act (2012) largely failed as they were devised in a manner that leaves the classification of NPS open to various justifications in structural and functional arguments, complicating legal and testing systems. A lack of consistent legislation that accurately and quickly determines which substances are deemed NPS contributes to this problem in the United States.

Although our analysis is comprehensive, it is not without limitations. First, samples were collected from cases limited to two geographic locations- Los Angeles and Sacramento. Although these two cities may represent a large and medium-sized city in California, they do not represent the rest of the cities and towns in the state. To a greater extent, they do not represent the rest of the United States. There are known geographic differences in drug use patterns so our findings may not accurately be used to extrapolate what may be true for the rest of the country. Furthermore, our cohort does not include subjects from other at-risk populations, such as drivers or pedestrians who may not have been involved in roadway crashes that required an Emergency Department visit. Certain exclusionary criteria, such as the patient having a warrant for their arrest also limit data extrapolation. Additionally, the study was conducted over 7 months, from January to July 2024. The general findings of our larger ongoing study demonstrated differential detection frequency among drugs between months. The detection frequency of most drugs, for example, was trending up towards the summer months (unpublished data). It is difficult to predict the impact that collection from August to December would have had on our data. This time constraint also makes it difficult to establish trends in NPS use over longer periods of time. Longitudinal studies would provide a more comprehensive understanding of NPS use and its evolving impact on impaired driving. Finally, like any other comprehensive LC-QTOF/MS method, our method may not have the best sensitivity for a few NPS. Comprehensive methods are designed to optimize detection and confirmation of as many drugs (NPS) as possible. However, the wide range of structural differences and polarities among various classes of NPS make it impossible to have optimum sensitivity for all NPS classes. Although our method is good at detecting more than 98% of our target analytes at sensitivities appropriate for serum, it does not have good sensitivity to very small, strongly polar (e.g., GHB analogs) and large highly hydrophobic (e.g., delta-9-THC derivatives) compounds, so if the levels of these NPS are low in biological samples, we likely missed their detection. Moreover, substances of which there is no known reference standard or are very uncommon might be excluded which may further limit the scope of the study.

In summary, although the number of cases where we found NPS in our cohort is limited, our findings provide more evidence of the ongoing danger that NPS may pose to road safety and highlight the need for policy change aimed at expanding NPS testing infrastructure, specifically in cases of impaired driving. Future research should continue to look at NPS in roadway collisions, specifically in drivers, as this is the cohort most responsible for injury and death in crashes. If possible, it would also be beneficial to develop a dynamic roadside testing strategy that adapts to the highly detected and impairing NPS reported in nationwide drug surveillance close to, if not in, real-time.

## Data Availability

The original contributions presented in the study are included in the article, further inquiries about the results presented can be directed to the corresponding authors.
